# Supplementary material for: Machine learning and natural language processing methods to identify ischemic stroke, acuity and location from radiology reports
Source: PLoS One. 2020 Jun 19;15(6):e0234908. doi: 10.1371/journal.pone.0234908 (PMC7304623; doi:10.1371/journal.pone.0234908)
Supplement: S1 File — (DOCX) [file pone.0234908.s001.docx]

**Supplementary Data:**

**eAppendix**

1. Example of Raw Text Report from the Derivation Cohort
2. Example of Raw Text Report from the Validation (BMC) Cohort
3. Description of Machine Learning Methods and Natural Language Processing (NLP) Featurization Techniques
   1. Bag of Words
   2. Term Frequency-Inverse Document Frequency (tf-idf)
   3. GloVe
4. Classification Methods
   1. Logistic Regression
   2. K-Nearest Neighbors (k-NN)
   3. CART
   4. Optimal Classification Trees (OCT)
   5. Random Forests
   6. Recurrent Neural Networks
5. Glove Online Link

**eTable 1:** Patient and Report Characteristics for the Derivation Cohort and the Validation (BMC) Cohort.

**eTable 2:** Percent Agreement between reports labeled by study-team members and Board Certified Neurologist and Intensivist.

**eTable 3:** Percent Agreement between reports labeled by study-team members and Board Certified Neurologist and Intensivist after raw image adjudication.

**eTable 4.** Sensitivity and Specificity Table for GLoVe and Classification Models.

**eTable 5.** McNemar Test Results for two best performing combinations of featurization technique and binary classification algorithm for all three tasks.

**eTable 6.** Performance Comparison of pre-trained Wikipedia GloVe embeddings with the proposed neurology specific embeddings using the RNN classifier across all three predictive tasks.

**eFigure 1.** GloVe vectors projected into a 2D space showing relationships between analogous pairs.

**eFigure 2.** Examples of OCT models with two partition nodes and three leaf nodes. The tree model at the left contains parallel splits whereas the one at the right includes hyperplane splits.

**eFigure 3.** Precision-Recall Curves for NLP methods.

**Supplemental References**

**eAppendix**

1. **Example of Raw Text Report from the Derivation Cohort (Identifying Information Redacted):**

Exam Number: XXXXXXXXXXX Report Status: Final Type: MR Brain w&w/oCont & FurthSequ Date/Time: XX/XX/XXX XX:XX Exam Code: M1234/GAD Ordering Provider: XXXX, XX, M.D. REPORT: HISTORY: Gait disturbance, lack of correlation, visual field deficit. Left hand clumsiness, left fissure tube. Recent left-sided CVA. Evaluation for acute right-sided infarction. TECHNIQUE: MRI of the brain is performed with sagittal T1, axial T1, T2, FLAIR, proton density, and diffusion weighted imaging. After administration IV contrast, axial T1 and coronal T1-weighted imaging are acquired. COMPARISON: MRI of the brain on XX/XX/XXXX. FINDINGS: Area of reduced diffusivity is present involving the right temporal lobe, parietal lobe, insula, and centrum semiovale, consistent with acute MCA infarction without hemorrhagic transformation or enhancement. In addition, multiple foci of reduced diffusivity is present in the corona radiata and bilateral cerebellum (left greater than right). One focal area in the left cerebellum exhibits subacute enhancement. No hemorrhagic transformation is seen. Patchy focal areas of T2 prolongation are present in the periventricular and deep white matter bilaterally, consistent with chronic microvascular ischemia. There is no mass-effect or midline shift. There is no evidence of hydrocephalus but the ventricle are prominent for the age. The paranasal sinuses and mastoid air cells are clear. There is asymmetric paucity of flow-related signal void in the right posterior circulation, which is unchanged. The paranasal sinuses and mastoid air cells are clear. IMPRESSION: 1. New acute right MCA infarction, probably less than one day old. 2. Evolving focal subacute infarctions in bilateral corona radiata and bilateral cerebellum (left greater than right), with a pattern compatible with embolic source. A focus in the left cerebellum shows subacute enhancement. No hemorrhagic transformation is seen. Discussed with the resident caring for the patient in the emergency department by Dr. XXXX XX/XX/XX roughly X:XX XX. RADIOLOGISTS: SIGNATURES: XXXXX, X MD(R)

1. **Example of Raw Text Report from the Validation (BMC) Cohort (Identifying Information Redacted):**

Exam Number: XXXXXXXXXXX Report Status: Final Type: CT HEAD WO CONTRAST Date/Time: XX/XX/XXX XX:XX Ordering Provider: XXXX, XX, M.D. REPORT: HISTORY: Hydrocephalus, obstructive TECHNIQUE: Contiguous 1.25 mm and 5 mm axial images of the head without intravenous contrast. Coronal and sagittal reformats. CT Dose Reduction: Exam was performed using either iterative reconstruction technique or adjustment of mA and/or kV according to the patient size. COMPARISON: CT brain: XX/XX/XXXX; MR outside: XX/XX/XXXX. FINDINGS: Since the prior study of XX/XX/XXXX there has been a decrease in the size of the lateral and third ventricles in this patient with a left EVD which terminates in the left frontal horn. There is now no significant dilatation of the ventricular system although there does persist some mass effect on the fourth ventricle and on the basilar cisterns in this patient who is status post left suboccipital decompressive craniectomy defect for evacuation of a left cerebellar infarct and hemorrhage. There is no definite tonsillar herniation now. It should be noted, however, that the patient is considerably rotated which limits evaluation. The pneumocephalus related to the left posterior fossa has decreased as has the mild low density axial/subgaleal collection and the soft tissue swelling adjacent to the left operative bed .There has also been some decrease in the degree of low density is seen in the left cerebellum likely representing some residual infarct and edema. The infarcts shown on the outside MR of XX/XX/XXXX in the right occipital and parietal lobes, the left occipital and left posterior temporal lobe and right and left cerebellum and brainstem are not seen to as good advantage on this plain CT scan. A nasogastric tube is seen in the right nasal cavity. Mild mucoperiosteal thickening is seen in the left maxillary and right sphenoid sinus. No significant opacification is seen in the mastoid air cells. Right frontal burr hole is seen for the EVD with adjacent scalp swelling. Some calcifications are again seen in the soft tissues inferior to the left occipital bone. Calcification in the carotid siphons are evident. The sellar is not enlarged. The orbits are grossly unremarkable. IMPRESSION: In this patient status post decompressive left suboccipital craniectomy for partial resection of a left cerebellar infarct and hemorrhage, the postoperative changes are decreasing as described above although there persists some mass effect on the fourth ventricle. Nevertheless, with the right EVD in place the ventricles are no longer dilated. Please see comments above in this patient with multiple recent infarcts supra and infratentorially.

1. **Description of Machine Learning Methods and Natural Language Processing (NLP) Featurization Techniques**
2. Bag of Words (BOW)

Bag of words is the simplest model for text featurization, disregarding context, semantic proximity and grammar. Each word in the main corpus/body of the text is considered a distinct feature. Every report can be represented with a *D*-dimensional vector, where *D* is equal to the vocabulary size found in the collective set of radiology reports. The value of each feature corresponds to the number of times a word was found in a given report. If the word was not present in the document, we assigned the value 0. For example, if the vocabulary size was 4,432, that means that each observation (report) would be encoded as 4,432 dimensional vector which would have positive values only for the words that were contained in its text. We also included 2-tuples of words to be included as a single feature in the case of common medical terminology.

1. Term Frequency-Inverse Document Frequency (tf-idf)

The term frequency-inverse document frequency method (tf-idf) builds upon the BOW framework by re-weighting the document features based on the relative importance of the word in the text.^2^ Weighting of words is positively correlated to the number of times a word appears in a given document, but is offset by frequency in the training corpus. Let $f_{t,d}$be the number of times term “$t$” appears in report “$d$” and “$s_{d}$” be the number of distinct words that appear in document $d$. We can then define the following:

- Term Frequency: $tf\left( t,d \right)= \frac{f_{t,d}}{s_{d}}$
- Inverse Document Frequency: $idf(t,d) = \log(\frac{N}{\sum_{d=1}^{N} \mathbb{l(}tf\left( t,d \right)>0)})$

where $N$ is the total number of documents. The latter term is a measure of how much information the word provides, i.e., if it is common or rare across all documents. Thus, we can define:

$$tf-idf\left( t,d,N \right)=tf\left( t,d \right)\cdot idf(t,d)$$

For example, consider the case of a report that includes 100 different terms wherein the word stroke is encountered 5 distinct times. The term frequency (“tf”) for stroke is then (5/100) = 0.05. Assuming 10 million documents and that the term “stroke” appears in 1,000 of these documents, the inverse document frequency (“idf”) is calculated as $\log\left( \frac{10,000,000}{1,000} \right)=4$. Thus, the tf-idf weight is the product of these quantities: $0.05*4=0.2$. This method does not consider the sequence of words in the text neither their semantic proximity. However, it is more successful in distinguishing the importance of words in the text based on their relative frequency.

1. GloVe

BOW and tf-idf are techniques for converting documents into structured numeric representations. It has become increasingly common in NLP to instead use word embeddings, which represent individual words as $d$-dimensional vectors and have been popularized through techniques like word2vec.^1^ While each dimension value in the vector does not have an absolute interpretation, word-embedding vectors allow for complex pairwise comparisons between words that capture underlying semantic relationships. See eFigure 1 for illustration.

A current and high-performing form of embedding is Global Vectors for Word Representation (GloVe).^2^ GloVe takes a corpus of text and looks at how often pairs of words co-occur in some window, since these frequencies have some sort of semantic meaning. For example, the pairs of terms “ice”-“solid” and “steam”-“gas” co-occur much more frequently than pairs “ice”-“gas” and “steam”-“solid.” Exact frequencies depend on the specific training corpus GloVe uses. The algorithm learns via a $d$-dimensional vector (usually $d$ is set between 100-300) for each word such that their dot product, a rough measure of how close they lie in the vector space, is a positively correlated function of the words' co-occurrence probability. These word representations then are either fed as inputs one-by-one into sequential models, or are converted into document representations by simply taking an average across words. We used the latter approach in all binary classifiers other than the RNN implementation.

The GloVe model parameters were set to the following: word vector dimension was selected to be 100, number of iterations was equal to 50, we used a window size of 10, and learning rate of 0.05.

1. **Classification Methods**
2. Logistic Regression

Logistic Regression is a simple yet very powerful classification. It is similar to the linear regression function but uses a nonlinear transformation to convert the output of the function to a probability. These probabilities are compared to a threshold value to predict a binary class. If one looks at the 'Logit' function (logarithm of the odds) the coefficients of the logistic response function can be interpreted in a similar fashion as those of the linear regression.

To improve the regression, we have added the “l1”/Lasso regularization term to protect it against feature-wise perturbations. This will ensure a greater robustness of the regression.^3^ We used 10-fold cross-validation to select the appropriate value of the regularization term λ, using a maximum of 1000 iterations. The tolerance threshold of the objective function was set to 0.01.

1. K-Nearest Neighbors (*k-NN*)

The $k$-Nearest Neighbors algorithm is a supervised technique that can be applied to both classification and regression problems.^4^ In a class prediction setting, given an observation in the testing set to be classified, the algorithm searches for $k$ observations in the labeled training set that are nearest in feature space, where $k$ is a small integer. The observation is then assigned to the class to which the majority of its neighbors belong. Though the $\boldsymbol{kNN}$ algorithm is the simplest of machine learning algorithms, it often has powerful empirical performance. Its simplicity is also an advantage in terms of interpretability – one can assess why a point was predicted to fall into a certain class by looking at its neighbors and in which features they are most similar. We used 10-fold cross-validation to select the appropriate value of the k parameter across the range of [5, 10, 15, 20].

1. CART

The Classification and Regression Trees (CART) methodology trains a decision tree by splitting variables with a greedy and top-down approach.^5^ The tree is built by branching on the value of a single variable after solving a local optimization problem but does not take into account previous splits. The tree starts with the root node and recurses on the resulting nodes. The algorithm stops when the predefined minimum number of observations per node is achieved. All the splits that do not decrease the impurity sufficiently are subsequently pruned to respect the maximum depth. CART has two major benefits: it does not assume a linear model and is interpretable as a result of the tree structure and its simple splits. To predict a class for an observation one has to follow the splits and at the end predict the most frequent outcome of the obtained leaf. We used 10-fold cross-validation to select the appropriate value of the minimum bucket and maximum depth parameters across the range of 1-10. The validation criterion was set to AUC.

1. Optimal Classification Trees (OCT)

Optimal Classification Trees (OCT) is an algorithm that trains highly accurate and interpretable classification decision-trees.^6^ Recently developed at MIT, this methodology leverages optimization techniques to construct the best decision tree for the training data in a single step. OCT differs from CART by solving for global optimality in the tree (as opposed to traditional greedy heuristics in which optimal decisions are made at each node without accounting for later nodes). Each node of the tree is split through a few high-importance variables in a straightforward manner, but is rebooted after each variable. Contrary to most of the modern high-accuracy but opaque ML techniques (e.g. neural networks and random forests), the tree structure of the OCT method retains interpretability.^7-9^ We used 10-fold cross-validation to select the appropriate value of the minimum bucket and maximum depth parameters across the range of 1-10. The validation criterion was selected in a similar process across the options of misclassification accuracy, gini, and entropy.

A variant of the OCT is the Optimal Classification Trees with Hyperplane (OCT-H) splits algorithm. While each split of the OCT is based on a single variable OCT-H authorizes multi-variable splits. This allows the algorithm to substantially improve its accuracy while only marginally impacting its interpretability. To better illustrated the concept of OCT and OCTH an example is displayed in eFigure 2. We used 10-fold cross-validation to select the appropriate value of the minimum bucket and maximum depth parameters across the ranges of 1-10 and 1-2 respectively. The validation criterion was selected in a similar process across the options of misclassification accuracy, gini, and entropy. The number of features considered in each split to form the hyperplane was selected between the values of [5, 10, 20, 30, 40, 50].

1. Random Forests

Random Forests is an ensemble machine learning method designed to improve the prediction accuracy of CART.^10^ It builds a large number of CART trees in parallel and combines them into a strong learner. Each CART tree only uses a random subset of the variables and is trained with a sample of the training data. The random forest makes its prediction by letting every trained tree vote and selects the outcome with the most votes. This technique works very well in practice as the combined findings of each individual tree uncover very complex patterns. Given the number of individual trees trained (in our case 500) this model’s interpretability decreases significantly. We used 10-fold cross-validation to select the appropriate value of the minimum bucket and maximum depth parameters across the range of 1-10. The validation criterion was set to AUC. The maximum number of greedy trees was set to 200.

1. Recurrent Neural Networks

Neural networks are computational nonlinear models that perform classification and regression tasks.^11^ Their key components are artificial “neurons” or processing elements which are organized in multiple interconnected layers including 1) An Input layer; 2) Hidden layers, and 3) An Output layer. Recurrent Neural Networks allow for back-propagation of the information in the model. This creates loops in the neural network architecture which act as a ‘memory state’ for its components. This state provides the neurons with the ability to account for the sequence in which information occurs.^12^ This structure has been particularly successful in Natural Language Processing applications where the sequence of words in the text can significantly impact the overall meaning of the corpus.^13^ We trained our models on a particular subclass of recurrent neural networks that utilize an efficient, gradient based method called Long Short-Term memory (LSTM).^14^

Our model includes two hidden layers. The first layer learns sentence vectors to represent semantics of sentences with an LSTM network, and in the second layer, the relations of sentences are encoded in document representation. We follow the structure suggested by Rao et al.^15^ We used 10-fold cross-validation and grid search to tune the hyperparameters of each model. Below you will find the range of values that were tested for each model:

Batch Size: 50, 100, 200

Number of Epochs: 50, 100, 200, 400

Size of the LSTM: 100, 500, 1000

Activation Function: sigmoid, ReLU

Input Layer Dropout: 0.1, 0.2, …,0.9

Recurrent Layer Dropout: 0.1, 0.2, …,0.9

Iterations: 2, 5, 10

1. **GloVe Online Link:**

Vector representations specifically tailored for neuroradiology purposes: [<http://www.mit.edu/~agniorf/files/Glove_Neurology_Embeddings.csv>]


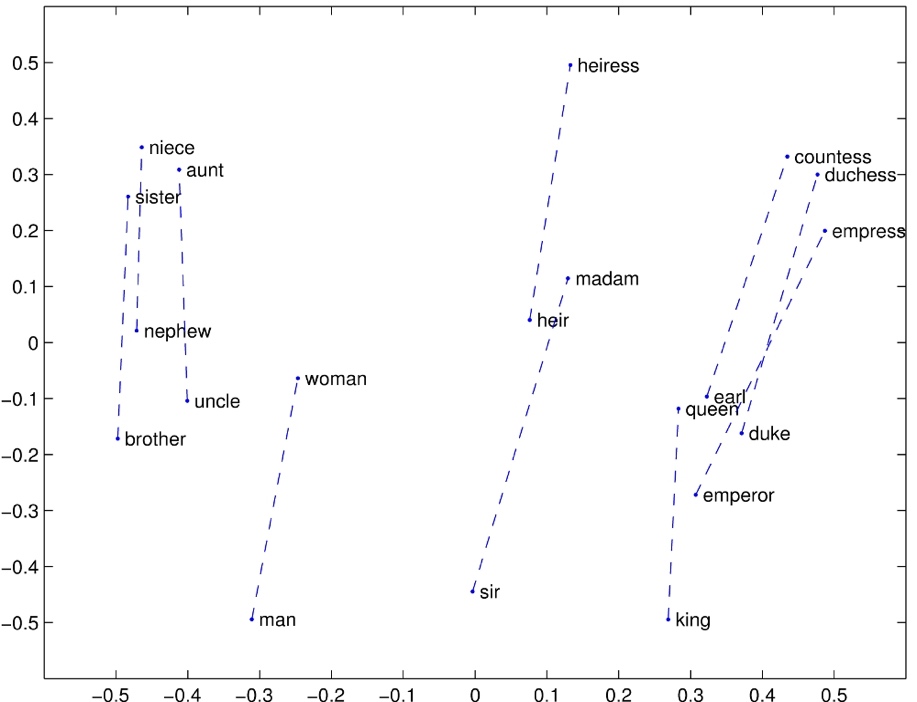
**eFigure 1.** GloVe vectors projected into a 2D space showing relationships between analogous pairs.^2^

**eFigure 2.** Examples of OCT models with two partition nodes and three leaf nodes. The tree model at the left contains parallel splits whereas the one at the right includes hyperplane splits.


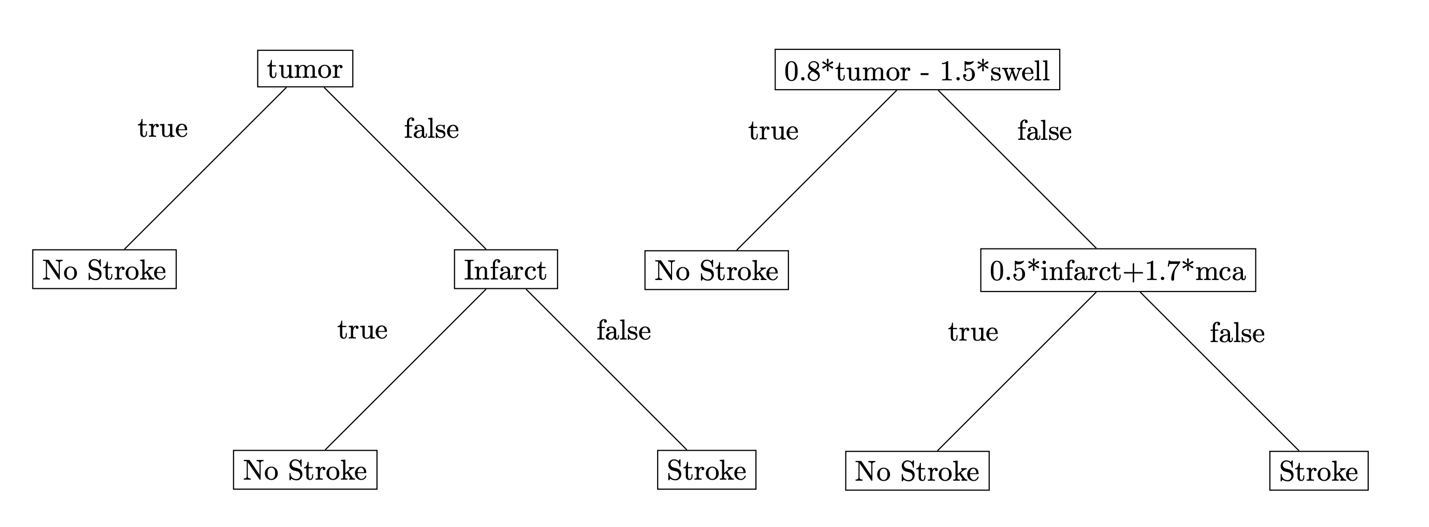


**eFigure 3.** Precision-Recall Curves for NLP classifications


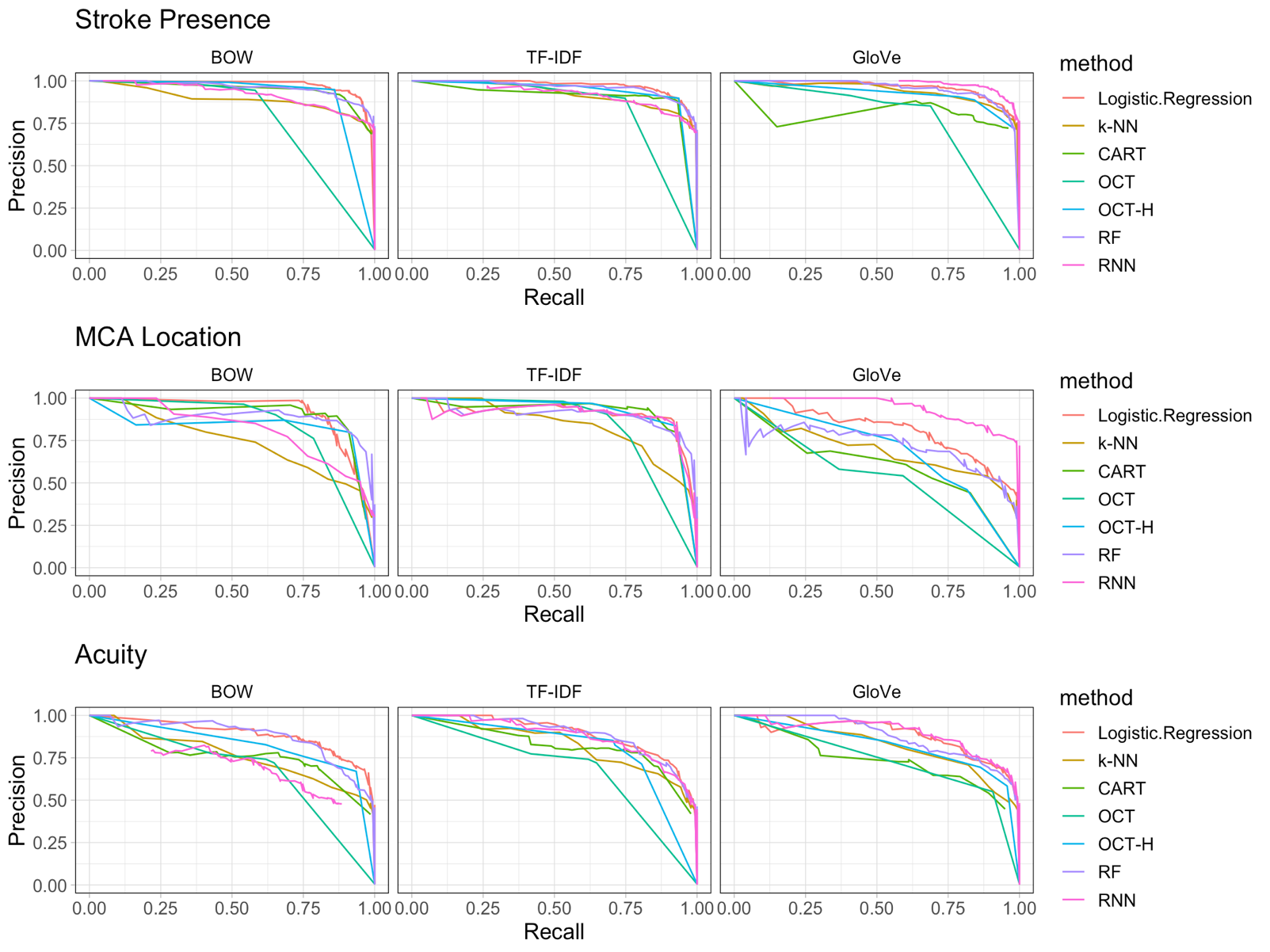


Precision Recall Curves for NLP classification of A) Stroke Presence, B) MCA location and C) Acuity using different combinations of text featurization (BOW, TF-IDF, GloVe) and binary classification algorithms (Logistic Regression, k-NN, CART, OCT, OCT-H, RF, RNN). GloVe and RNN achieved the best performance for all three tasks (>90%). Similar results were achieved for simple tasks by BOW or TF-IDF paired with Logistic Regression. The results presented average the mean recall and precision over five random splits of the data.

**eTable 1:** Patient and Report Characteristics for the Derivation (PARTNER’S) and Validation (BMC) Cohorts.

|  | |  |  |
| --- | --- | --- | --- |
| **Derivation (PARTNER’S) Cohort** | | **Validation (BMC) Cohort^†^** | |
| Characteristics | n (%) | Characteristics | n (%) |
| Patients | 297 | Patients | 424 |
| Total Reports | 1359 | Total Reports | 500 |
| Stroke Reports | 925 | Stroke Reports | 266 |
| MCA Territory Involvement Reports | 350 | MCA Territory Involvement Reports | 90 |
| Acute Stroke Reports | 522 | Acute Stroke Reports | 106 |
| Reports per Patient (median, [IQR]) | 7 [3,11] | Reports per Patient (median, [IQR]) | 1.79 [1,3] |
| Sex |  | Sex |  |
| Female | 129 (43) | Female | 192 (45) |
| Male | 168 (57) | Male | 229 (54) |
| Age (median, [IQR]) | 68 [55,79] | Age (median, [IQR]) | 69 [59,79] |
| Race |  | Race |  |
| White | 230 (77.4) | White | 125 (29.5) |
| Black or African American | 22 (7.4) | Black or African American | 184 (43.4) |
| Asian | 13 (4.4) | Asian | 23 (5.4) |
| Hispanic or Latino | 11 (3.7) | Hispanic or Latino | 51 (12) |
| American Indian or Alaska Native | 1 (0.3) | American Indian or Alaska Native | 0 (0) |
| Other/Unknown | 20 (6.7) | Other/Unknown | 38 (8.3) |
| Average report length (words) | 264.7 | Average report length (words) | 304.3 |
| Median report length (words) | 215.0 | Median report length (words) | 261.5 |
| Average report length (characters) | 1903.1 | Average report length (characters) | 2135.6 |
| Median report length (characters) | 1559.0 | Median report length (characters) | 1831.5 |

**^†^**Demographic data was missing for 3 patients; percentages still reflect the entire cohort.

**eTable 2:** Percent Agreement between reports labeled by study-team members and Board Certified Neurologist and Intensivist.

| **Derivation + Validation Cohort**  **n = 150** | |
| --- | --- |
| Stroke | 90.7% |
| MCA Territory Involvement | 87.3% |
| Acuity | 93.3% |

**eTable 3:** Percent Agreement between reports labeled by study-team members and Board Certified Neurologist and Intensivist after raw image adjudication.

|  | **Derivation Cohort**  **n = 300** |
| --- | --- |
| **Stroke** | 96.7% |
| **MCA Territory Involvement** | 95.3% |
| **Acuity** | 98.0% |

**eTable 4:** Sensitivity and Specificity Table for GLoVe and Classification Models.

|  |  | |  | |  |  |  | | |  |
| --- | --- | --- | --- | --- | --- | --- | --- | --- | --- | --- |
| **Threshold** | **Accuracy** | **Sensitivity** | | **Specificity** | **Method** | **Embedding** | | **Task** |  |  |
| 0.1 | 0.6882 | 0.9915 | | 0.0189 | CART | BOW | | stroke |  |  |
| 0.25 | 0.8588 | 0.8932 | | 0.7830 | CART | BOW | | stroke |  |  |
| 0.75 | 0.8265 | 0.7949 | | 0.8962 | CART | BOW | | stroke |  |  |
| 0.9 | 0.7735 | 0.7051 | | 0.9245 | CART | BOW | | stroke |  |  |
| 0.1 | 0.3118 | 1.0000 | | 0.0000 | k-NN | BOW | | stroke |  |  |
| 0.25 | 0.6882 | 0.9957 | | 0.0094 | k-NN | BOW | | stroke |  |  |
| 0.75 | 0.7735 | 0.8718 | | 0.5566 | k-NN | BOW | | stroke |  |  |
| 0.9 | 0.5294 | 0.3590 | | 0.9057 | k-NN | BOW | | stroke |  |  |
| 0.1 | 0.8118 | 0.9701 | | 0.4623 | Logistic Regression | BOW | | stroke |  |  |
| 0.25 | 0.8676 | 0.9573 | | 0.6698 | Logistic Regression | BOW | | stroke |  |  |
| 0.75 | 0.8824 | 0.8803 | | 0.8868 | Logistic Regression | BOW | | stroke |  |  |
| 0.9 | 0.8618 | 0.8248 | | 0.9434 | Logistic Regression | BOW | | stroke |  |  |
| 0.1 | 0.3118 | 1.0000 | | 0.0000 | RF | BOW | | stroke |  |  |
| 0.25 | 0.6912 | 1.0000 | | 0.0094 | RF | BOW | | stroke |  |  |
| 0.75 | 0.7147 | 0.6068 | | 0.9528 | RF | BOW | | stroke |  |  |
| 0.9 | 0.3529 | 0.0598 | | 1.0000 | RF | BOW | | stroke |  |  |
| 0.1 | 0.3118 | 1.0000 | | 0.0000 | OCT | BOW | | stroke |  |  |
| 0.25 | 0.3118 | 1.0000 | | 0.0000 | OCT | BOW | | stroke |  |  |
| 0.75 | 0.6882 | 0.5812 | | 0.9245 | OCT | BOW | | stroke |  |  |
| 0.9 | 0.6882 | 0.5812 | | 0.9245 | OCT | BOW | | stroke |  |  |
| 0.1 | 0.3118 | 1.0000 | | 0.0000 | OCT-H | BOW | | stroke |  |  |
| 0.25 | 0.3118 | 1.0000 | | 0.0000 | OCT-H | BOW | | stroke |  |  |
| 0.75 | 0.8706 | 0.8590 | | 0.8962 | OCT-H | BOW | | stroke |  |  |
| 0.9 | 0.8706 | 0.8590 | | 0.8962 | OCT-H | BOW | | stroke |  |  |
| 0.1 | 0.3118 | 1.0000 | | 0.0000 | RNN | BOW | | stroke |  |  |
| 0.25 | 0.7000 | 1.0000 | | 0.0377 | RNN | BOW | | stroke |  |  |
| 0.75 | 0.6588 | 0.5470 | | 0.9057 | RNN | BOW | | stroke |  |  |
| 0.9 | 0.4618 | 0.2222 | | 0.9906 | RNN | BOW | | stroke |  |  |
| 0.1 | 0.3118 | 1.0000 | | 0.0000 | CART | TF-IDF | | stroke |  |  |
| 0.25 | 0.8647 | 0.9316 | | 0.7170 | CART | TF-IDF | | stroke |  |  |
| 0.75 | 0.8059 | 0.7949 | | 0.8302 | CART | TF-IDF | | stroke |  |  |
| 0.9 | 0.7794 | 0.7521 | | 0.8396 | CART | TF-IDF | | stroke |  |  |
| 0.1 | 0.6912 | 1.0000 | | 0.0094 | k-NN | TF-IDF | | stroke |  |  |
| 0.25 | 0.7000 | 0.9957 | | 0.0472 | k-NN | TF-IDF | | stroke |  |  |
| **Threshold** | **Accuracy** | **Sensitivity** | | **Specificity** | **Method** | **Embedding** | | **Task** |  |  |
| 0.75 | 0.7794 | 0.8376 | | 0.6509 | k-NN | TF-IDF | | stroke |  |  |
| 0.9 | 0.5971 | 0.4274 | | 0.9717 | k-NN | TF-IDF | | stroke |  |  |
| 0.1 | 0.3118 | 1.0000 | | 0.0000 | Logistic Regression | TF-IDF | | stroke |  |  |
| 0.25 | 0.7176 | 0.9957 | | 0.1038 | Logistic Regression | TF-IDF | | stroke |  |  |
| 0.75 | 0.8118 | 0.7436 | | 0.9623 | Logistic Regression | TF-IDF | | stroke |  |  |
| 0.9 | 0.5794 | 0.3889 | | 1.0000 | Logistic Regression | TF-IDF | | stroke |  |  |
| 0.1 | 0.3118 | 1.0000 | | 0.0000 | RF | TF-IDF | | stroke |  |  |
| 0.25 | 0.6912 | 1.0000 | | 0.0094 | RF | TF-IDF | | stroke |  |  |
| 0.75 | 0.6971 | 0.5769 | | 0.9623 | RF | TF-IDF | | stroke |  |  |
| 0.9 | 0.3618 | 0.0726 | | 1.0000 | RF | TF-IDF | | stroke |  |  |
| 0.1 | 0.3118 | 1.0000 | | 0.0000 | OCT | TF-IDF | | stroke |  |  |
| 0.25 | 0.3118 | 1.0000 | | 0.0000 | OCT | TF-IDF | | stroke |  |  |
| 0.75 | 0.7676 | 0.7479 | | 0.8113 | OCT | TF-IDF | | stroke |  |  |
| 0.9 | 0.5382 | 0.3333 | | 0.9906 | OCT | TF-IDF | | stroke |  |  |
| 0.1 | 0.3118 | 1.0000 | | 0.0000 | OCT-H | TF-IDF | | stroke |  |  |
| 0.25 | 0.8824 | 0.9359 | | 0.7642 | OCT-H | TF-IDF | | stroke |  |  |
| 0.75 | 0.8824 | 0.9359 | | 0.7642 | OCT-H | TF-IDF | | stroke |  |  |
| 0.9 | 0.6941 | 0.5726 | | 0.9623 | OCT-H | TF-IDF | | stroke |  |  |
| 0.1 | 0.7059 | 0.9915 | | 0.0755 | RNN | TF-IDF | | stroke |  |  |
| 0.25 | 0.7471 | 0.9744 | | 0.2453 | RNN | TF-IDF | | stroke |  |  |
| 0.75 | 0.7000 | 0.6154 | | 0.8868 | RNN | TF-IDF | | stroke |  |  |
| 0.9 | 0.5676 | 0.3932 | | 0.9528 | RNN | TF-IDF | | stroke |  |  |
| 0.1 | 0.7206 | 0.9145 | | 0.2925 | CART | GloVe | | stroke |  |  |
| 0.25 | 0.7118 | 0.8504 | | 0.4057 | CART | GloVe | | stroke |  |  |
| 0.75 | 0.7176 | 0.7009 | | 0.7547 | CART | GloVe | | stroke |  |  |
| 0.9 | 0.6912 | 0.6368 | | 0.8113 | CART | GloVe | | stroke |  |  |
| 0.1 | 0.3118 | 1.0000 | | 0.0000 | k-NN | GloVe | | stroke |  |  |
| 0.25 | 0.7000 | 1.0000 | | 0.0377 | k-NN | GloVe | | stroke |  |  |
| 0.75 | 0.8147 | 0.8504 | | 0.7358 | k-NN | GloVe | | stroke |  |  |
| 0.9 | 0.6294 | 0.4701 | | 0.9811 | k-NN | GloVe | | stroke |  |  |
| 0.1 | 0.7147 | 1.0000 | | 0.0849 | Logistic Regression | GloVe | | stroke |  |  |
| 0.25 | 0.7706 | 0.9829 | | 0.3019 | Logistic Regression | GloVe | | stroke |  |  |
| 0.75 | 0.7971 | 0.7350 | | 0.9340 | Logistic Regression | GloVe | | stroke |  |  |
|  | | | | | | | | |  |  |
| **Threshold** | **Accuracy** | **Sensitivity** | | **Specificity** | **Method** | **Embedding** | | **Task** |  |  |
| 0.9 | 0.6353 | 0.4744 | | 0.9906 | Logistic Regression | GloVe | | stroke |  |  |
| 0.1 | 0.3118 | 1.0000 | | 0.0000 | RF | GloVe | | stroke |  |  |
| 0.25 | 0.7000 | 0.9829 | | 0.0755 | RF | GloVe | | stroke |  |  |
| 0.75 | 0.7471 | 0.6624 | | 0.9340 | RF | GloVe | | stroke |  |  |
| 0.9 | 0.5029 | 0.2778 | | 1.0000 | RF | GloVe | | stroke |  |  |
| 0.1 | 0.3118 | 1.0000 | | 0.0000 | OCT | GloVe | | stroke |  |  |
| 0.25 | 0.3118 | 1.0000 | | 0.0000 | OCT | GloVe | | stroke |  |  |
| 0.75 | 0.6206 | 0.5256 | | 0.8302 | OCT | GloVe | | stroke |  |  |
| 0.9 | 0.5647 | 0.4060 | | 0.9151 | OCT | GloVe | | stroke |  |  |
| 0.1 | 0.7294 | 0.9744 | | 0.1887 | OCT-H | GloVe | | stroke |  |  |
| 0.25 | 0.7294 | 0.9744 | | 0.1887 | OCT-H | GloVe | | stroke |  |  |
| 0.75 | 0.7971 | 0.7863 | | 0.8208 | OCT-H | GloVe | | stroke |  |  |
| 0.9 | 0.7971 | 0.7863 | | 0.8208 | OCT-H | GloVe | | stroke |  |  |
| 0.1 | 0.7147 | 1.0000 | | 0.0849 | RNN | GloVe | | stroke |  |  |
| 0.25 | 0.7824 | 1.0000 | | 0.3019 | RNN | GloVe | | stroke |  |  |
| 0.75 | 0.8912 | 0.8718 | | 0.9340 | RNN | GloVe | | stroke |  |  |
| 0.9 | 0.8059 | 0.7222 | | 0.9906 | RNN | GloVe | | stroke |  |  |
| 0.1 | 0.9118 | 0.9082 | | 0.9132 | CART | BOW | | location |  |  |
| 0.25 | 0.9324 | 0.8673 | | 0.9587 | CART | BOW | | location |  |  |
| 0.75 | 0.9118 | 0.7449 | | 0.9793 | CART | BOW | | location |  |  |
| 0.9 | 0.9059 | 0.7041 | | 0.9876 | CART | BOW | | location |  |  |
| 0.1 | 0.4324 | 0.9796 | | 0.2107 | k-NN | BOW | | location |  |  |
| 0.25 | 0.6559 | 0.9490 | | 0.5372 | k-NN | BOW | | location |  |  |
| 0.75 | 0.7324 | 0.0714 | | 1.0000 | k-NN | BOW | | location |  |  |
| 0.9 | 0.7118 | 0.0000 | | 1.0000 | k-NN | BOW | | location |  |  |
| 0.1 | 0.8588 | 0.8980 | | 0.8430 | Logistic Regression | BOW | | location |  |  |
| 0.25 | 0.9088 | 0.8469 | | 0.9339 | Logistic Regression | BOW | | location |  |  |
| 0.75 | 0.9176 | 0.7653 | | 0.9793 | Logistic Regression | BOW | | location |  |  |
| 0.9 | 0.9176 | 0.7245 | | 0.9959 | Logistic Regression | BOW | | location |  |  |
| 0.1 | 0.7118 | 1.0000 | | 0.0000 | RF | BOW | | location |  |  |
| 0.25 | 0.8794 | 0.9490 | | 0.8512 | RF | BOW | | location |  |  |
| 0.75 | 0.7118 | 0.0000 | | 1.0000 | RF | BOW | | location |  |  |
| 0.9 | 0.7118 | 0.0000 | | 1.0000 | RF | BOW | | location |  |  |
| 0.1 | 0.8676 | 0.7857 | | 0.9008 | OCT | BOW | | location |  |  |
| 0.25 | 0.8676 | 0.7857 | | 0.9008 | OCT | BOW | | location |  |  |
| 0.75 | 0.8618 | 0.5408 | | 0.9917 | OCT | BOW | | location |  |  |
| **Threshold** | **Accuracy** | **Sensitivity** | | **Specificity** | **Method** | **Embedding** | | **Task** |  |  |
| 0.9 | 0.7118 | 0.0000 | | 1.0000 | OCT | BOW | | location |  |  |
| 0.1 | 0.9088 | 0.9184 | | 0.9050 | OCT-H | BOW | | location |  |  |
| 0.25 | 0.9088 | 0.9184 | | 0.9050 | OCT-H | BOW | | location |  |  |
| 0.75 | 0.8794 | 0.6837 | | 0.9587 | OCT-H | BOW | | location |  |  |
| 0.9 | 0.8794 | 0.6837 | | 0.9587 | OCT-H | BOW | | location |  |  |
| 0.1 | 0.4353 | 0.9898 | | 0.2107 | RNN | BOW | | location |  |  |
| 0.25 | 0.6588 | 0.9592 | | 0.5372 | RNN | BOW | | location |  |  |
| 0.75 | 0.7441 | 0.1122 | | 1.0000 | RNN | BOW | | location |  |  |
| 0.9 | 0.7147 | 0.0102 | | 1.0000 | RNN | BOW | | location |  |  |
| 0.1 | 0.9118 | 0.9286 | | 0.9050 | CART | TF-IDF | | location |  |  |
| 0.25 | 0.9324 | 0.8265 | | 0.9752 | CART | TF-IDF | | location |  |  |
| 0.75 | 0.9176 | 0.7551 | | 0.9835 | CART | TF-IDF | | location |  |  |
| 0.9 | 0.9059 | 0.7143 | | 0.9835 | CART | TF-IDF | | location |  |  |
| 0.1 | 0.3647 | 1.0000 | | 0.1074 | k-NN | TF-IDF | | location |  |  |
| 0.25 | 0.6618 | 0.9694 | | 0.5372 | k-NN | TF-IDF | | location |  |  |
| 0.75 | 0.7382 | 0.0918 | | 1.0000 | k-NN | TF-IDF | | location |  |  |
| 0.9 | 0.7147 | 0.0102 | | 1.0000 | k-NN | TF-IDF | | location |  |  |
| 0.1 | 0.7853 | 0.9694 | | 0.7107 | Logistic Regression | TF-IDF | | location |  |  |
| 0.25 | 0.9265 | 0.9286 | | 0.9256 | Logistic Regression | TF-IDF | | location |  |  |
| 0.75 | 0.8500 | 0.5000 | | 0.9917 | Logistic Regression | TF-IDF | | location |  |  |
| 0.9 | 0.7912 | 0.2959 | | 0.9917 | Logistic Regression | TF-IDF | | location |  |  |
| 0.1 | 0.7118 | 1.0000 | | 0.0000 | RF | TF-IDF | | location |  |  |
| 0.25 | 0.8882 | 0.9388 | | 0.8678 | RF | TF-IDF | | location |  |  |
| 0.75 | 0.7118 | 0.0000 | | 1.0000 | RF | TF-IDF | | location |  |  |
| 0.9 | 0.7118 | 0.0000 | | 1.0000 | RF | TF-IDF | | location |  |  |
| 0.1 | 0.8647 | 0.7653 | | 0.9050 | OCT | TF-IDF | | location |  |  |
| 0.25 | 0.8647 | 0.7653 | | 0.9050 | OCT | TF-IDF | | location |  |  |
| 0.75 | 0.8618 | 0.5306 | | 0.9959 | OCT | TF-IDF | | location |  |  |
| 0.9 | 0.7118 | 0.0000 | | 1.0000 | OCT | TF-IDF | | location |  |  |
| 0.1 | 0.9265 | 0.9286 | | 0.9256 | OCT-H | TF-IDF | | location |  |  |
| 0.25 | 0.9265 | 0.9286 | | 0.9256 | OCT-H | TF-IDF | | location |  |  |
| 0.75 | 0.8882 | 0.6327 | | 0.9917 | OCT-H | TF-IDF | | location |  |  |
| 0.9 | 0.8882 | 0.6327 | | 0.9917 | OCT-H | TF-IDF | | location |  |  |
| 0.1 | 0.7412 | 0.9694 | | 0.6488 | RNN | TF-IDF | | location |  |  |
| 0.25 | 0.9294 | 0.9286 | | 0.9298 | RNN | TF-IDF | | location |  |  |
| 0.75 | 0.8529 | 0.5102 | | 0.9917 | RNN | TF-IDF | | location |  |  |
| **Threshold** | **Accuracy** | **Sensitivity** | | **Specificity** | **Method** | **Embedding** | | **Task** |  |  |
| 0.9 | 0.7912 | 0.2959 | | 0.9917 | RNN | TF-IDF | | location |  |  |
| 0.1 | 0.6500 | 0.8265 | | 0.5785 | CART | GloVe | | location |  |  |
| 0.25 | 0.7735 | 0.6020 | | 0.8430 | CART | GloVe | | location |  |  |
| 0.75 | 0.7500 | 0.2551 | | 0.9504 | CART | GloVe | | location |  |  |
| 0.9 | 0.7118 | 0.0000 | | 1.0000 | CART | GloVe | | location |  |  |
| 0.1 | 0.4265 | 0.9898 | | 0.1983 | k-NN | GloVe | | location |  |  |
| 0.25 | 0.7176 | 0.9082 | | 0.6405 | k-NN | GloVe | | location |  |  |
| 0.75 | 0.7176 | 0.0204 | | 1.0000 | k-NN | GloVe | | location |  |  |
| 0.9 | 0.7118 | 0.0000 | | 1.0000 | k-NN | GloVe | | location |  |  |
| 0.1 | 0.6088 | 0.9898 | | 0.4545 | Logistic Regression | GloVe | | location |  |  |
| 0.25 | 0.8059 | 0.8469 | | 0.7893 | Logistic Regression | GloVe | | location |  |  |
| 0.75 | 0.7618 | 0.1735 | | 1.0000 | Logistic Regression | GloVe | | location |  |  |
| 0.9 | 0.7235 | 0.0408 | | 1.0000 | Logistic Regression | GloVe | | location |  |  |
| 0.1 | 0.4647 | 0.9898 | | 0.2521 | RF | GloVe | | location |  |  |
| 0.25 | 0.7676 | 0.8469 | | 0.7355 | RF | GloVe | | location |  |  |
| 0.75 | 0.7235 | 0.0408 | | 1.0000 | RF | GloVe | | location |  |  |
| 0.9 | 0.7118 | 0.0000 | | 1.0000 | RF | GloVe | | location |  |  |
| 0.1 | 0.7118 | 1.0000 | | 0.0000 | OCT | GloVe | | location |  |  |
| 0.25 | 0.7382 | 0.5918 | | 0.7975 | OCT | GloVe | | location |  |  |
| 0.75 | 0.7118 | 0.0000 | | 1.0000 | OCT | GloVe | | location |  |  |
| 0.9 | 0.7118 | 0.0000 | | 1.0000 | OCT | GloVe | | location |  |  |
| 0.1 | 0.6706 | 0.8163 | | 0.6116 | OCT-H | GloVe | | location |  |  |
| 0.25 | 0.7324 | 0.7347 | | 0.7314 | OCT-H | GloVe | | location |  |  |
| 0.75 | 0.8206 | 0.5816 | | 0.9174 | OCT-H | GloVe | | location |  |  |
| 0.9 | 0.7118 | 0.0000 | | 1.0000 | OCT-H | GloVe | | location |  |  |
| 0.1 | 0.6118 | 1.0000 | | 0.4545 | RNN | GloVe | | location |  |  |
| 0.25 | 0.8500 | 1.0000 | | 0.7893 | RNN | GloVe | | location |  |  |
| 0.75 | 0.8706 | 0.5510 | | 1.0000 | RNN | GloVe | | location |  |  |
| 0.9 | 0.7912 | 0.2755 | | 1.0000 | RNN | GloVe | | location |  |  |
| 0.1 | 0.7647 | 0.8058 | | 0.7363 | CART | BOW | | acuity |  |  |
| 0.25 | 0.7794 | 0.7986 | | 0.7662 | CART | BOW | | acuity |  |  |
| 0.75 | 0.7235 | 0.4460 | | 0.9154 | CART | BOW | | acuity |  |  |
| 0.9 | 0.6912 | 0.3525 | | 0.9254 | CART | BOW | | acuity |  |  |
| 0.1 | 0.4824 | 0.9928 | | 0.1294 | k-NN | BOW | | acuity |  |  |
| 0.25 | 0.5588 | 0.9856 | | 0.2637 | k-NN | BOW | | acuity |  |  |
| 0.75 | 0.6559 | 0.1871 | | 0.9801 | k-NN | BOW | | acuity |  |  |
| **Threshold** | **Accuracy** | **Sensitivity** | | **Specificity** | **Method** | **Embedding** | | **Task** |  |  |
| 0.9 | 0.6118 | 0.0504 | | 1.0000 | k-NN | BOW | | acuity |  |  |
| 0.1 | 0.7853 | 0.9784 | | 0.6517 | Logistic Regression | BOW | | acuity |  |  |
| 0.25 | 0.8353 | 0.8777 | | 0.8060 | Logistic Regression | BOW | | acuity |  |  |
| 0.75 | 0.8176 | 0.6475 | | 0.9353 | Logistic Regression | BOW | | acuity |  |  |
| 0.9 | 0.7941 | 0.5468 | | 0.9652 | Logistic Regression | BOW | | acuity |  |  |
| 0.1 | 0.5912 | 1.0000 | | 0.0000 | RF | BOW | | acuity |  |  |
| 0.25 | 0.6735 | 0.9640 | | 0.4726 | RF | BOW | | acuity |  |  |
| 0.75 | 0.6294 | 0.1007 | | 0.9950 | RF | BOW | | acuity |  |  |
| 0.9 | 0.5912 | 0.0000 | | 1.0000 | RF | BOW | | acuity |  |  |
| 0.1 | 0.5912 | 1.0000 | | 0.0000 | OCT | BOW | | acuity |  |  |
| 0.25 | 0.7559 | 0.6187 | | 0.8507 | OCT | BOW | | acuity |  |  |
| 0.75 | 0.5912 | 0.0000 | | 1.0000 | OCT | BOW | | acuity |  |  |
| 0.9 | 0.5912 | 0.0000 | | 1.0000 | OCT | BOW | | acuity |  |  |
| 0.1 | 0.7853 | 0.9353 | | 0.6816 | OCT-H | BOW | | acuity |  |  |
| 0.25 | 0.7853 | 0.9353 | | 0.6816 | OCT-H | BOW | | acuity |  |  |
| 0.75 | 0.7912 | 0.6187 | | 0.9104 | OCT-H | BOW | | acuity |  |  |
| 0.9 | 0.5912 | 0.0000 | | 1.0000 | OCT-H | BOW | | acuity |  |  |
| 0.1 | 0.5912 | 0.8561 | | 0.4080 | RNN | BOW | | acuity |  |  |
| 0.25 | 0.6412 | 0.7698 | | 0.5522 | RNN | BOW | | acuity |  |  |
| 0.75 | 0.7206 | 0.4532 | | 0.9055 | RNN | BOW | | acuity |  |  |
| 0.9 | 0.6882 | 0.3237 | | 0.9403 | RNN | BOW | | acuity |  |  |
| 0.1 | 0.7971 | 0.8417 | | 0.7662 | CART | TF-IDF | | acuity |  |  |
| 0.25 | 0.7971 | 0.8417 | | 0.7662 | CART | TF-IDF | | acuity |  |  |
| 0.75 | 0.7618 | 0.5612 | | 0.9005 | CART | TF-IDF | | acuity |  |  |
| 0.9 | 0.7265 | 0.4173 | | 0.9403 | CART | TF-IDF | | acuity |  |  |
| 0.1 | 0.4529 | 1.0000 | | 0.0746 | k-NN | TF-IDF | | acuity |  |  |
| 0.25 | 0.5706 | 0.9640 | | 0.2985 | k-NN | TF-IDF | | acuity |  |  |
| 0.75 | 0.7206 | 0.3453 | | 0.9801 | k-NN | TF-IDF | | acuity |  |  |
| 0.9 | 0.6029 | 0.0288 | | 1.0000 | k-NN | TF-IDF | | acuity |  |  |
| 0.1 | 0.5029 | 0.9928 | | 0.1642 | Logistic Regression | TF-IDF | | acuity |  |  |
| 0.25 | 0.7382 | 0.9424 | | 0.5970 | Logistic Regression | TF-IDF | | acuity |  |  |
| 0.75 | 0.7176 | 0.3165 | | 0.9950 | Logistic Regression | TF-IDF | | acuity |  |  |
| 0.9 | 0.6265 | 0.0863 | | 1.0000 | Logistic Regression | TF-IDF | | acuity |  |  |
| 0.1 | 0.4206 | 1.0000 | | 0.0199 | RF | TF-IDF | | acuity |  |  |
| **Threshold** | **Accuracy** | **Sensitivity** | | **Specificity** | **Method** | **Embedding** | | **Task** |  |  |
| 0.25 | 0.6647 | 0.9640 | | 0.4577 | RF | TF-IDF | | acuity |  |  |
| 0.75 | 0.6235 | 0.0791 | | 1.0000 | RF | TF-IDF | | acuity |  |  |
| 0.9 | 0.5912 | 0.0000 | | 1.0000 | RF | TF-IDF | | acuity |  |  |
| 0.1 | 0.5912 | 1.0000 | | 0.0000 | OCT | TF-IDF | | acuity |  |  |
| 0.25 | 0.7559 | 0.6187 | | 0.8507 | OCT | TF-IDF | | acuity |  |  |
| 0.75 | 0.5912 | 0.0000 | | 1.0000 | OCT | TF-IDF | | acuity |  |  |
| 0.9 | 0.5912 | 0.0000 | | 1.0000 | OCT | TF-IDF | | acuity |  |  |
| 0.1 | 0.7882 | 0.8058 | | 0.7761 | OCT-H | TF-IDF | | acuity |  |  |
| 0.25 | 0.8206 | 0.7122 | | 0.8955 | OCT-H | TF-IDF | | acuity |  |  |
| 0.75 | 0.8206 | 0.7122 | | 0.8955 | OCT-H | TF-IDF | | acuity |  |  |
| 0.9 | 0.8294 | 0.7050 | | 0.9154 | OCT-H | TF-IDF | | acuity |  |  |
| 0.1 | 0.4412 | 1.0000 | | 0.0547 | RNN | TF-IDF | | acuity |  |  |
| 0.25 | 0.6588 | 0.9640 | | 0.4478 | RNN | TF-IDF | | acuity |  |  |
| 0.75 | 0.6265 | 0.0863 | | 1.0000 | RNN | TF-IDF | | acuity |  |  |
| 0.9 | 0.5912 | 0.0000 | | 1.0000 | RNN | TF-IDF | | acuity |  |  |
| 0.1 | 0.5000 | 0.9496 | | 0.1891 | CART | GloVe | | acuity |  |  |
| 0.25 | 0.7294 | 0.7554 | | 0.7114 | CART | GloVe | | acuity |  |  |
| 0.75 | 0.6824 | 0.2950 | | 0.9502 | CART | GloVe | | acuity |  |  |
| 0.9 | 0.6794 | 0.2590 | | 0.9701 | CART | GloVe | | acuity |  |  |
| 0.1 | 0.5235 | 1.0000 | | 0.1940 | k-NN | GloVe | | acuity |  |  |
| 0.25 | 0.6088 | 0.9640 | | 0.3632 | k-NN | GloVe | | acuity |  |  |
| 0.75 | 0.6647 | 0.1799 | | 1.0000 | k-NN | GloVe | | acuity |  |  |
| 0.9 | 0.6000 | 0.0216 | | 1.0000 | k-NN | GloVe | | acuity |  |  |
| 0.1 | 0.5500 | 0.9928 | | 0.2438 | Logistic Regression | GloVe | | acuity |  |  |
| 0.25 | 0.7647 | 0.9568 | | 0.6318 | Logistic Regression | GloVe | | acuity |  |  |
| 0.75 | 0.7382 | 0.3741 | | 0.9900 | Logistic Regression | GloVe | | acuity |  |  |
| 0.9 | 0.6235 | 0.0791 | | 1.0000 | Logistic Regression | GloVe | | acuity |  |  |
| 0.1 | 0.4824 | 1.0000 | | 0.1244 | RF | GloVe | | acuity |  |  |
| 0.25 | 0.7029 | 0.9784 | | 0.5124 | RF | GloVe | | acuity |  |  |
| 0.75 | 0.6735 | 0.2014 | | 1.0000 | RF | GloVe | | acuity |  |  |
| 0.9 | 0.6088 | 0.0432 | | 1.0000 | RF | GloVe | | acuity |  |  |
| 0.1 | 0.5912 | 1.0000 | | 0.0000 | OCT | GloVe | | acuity |  |  |
| 0.25 | 0.6588 | 0.9065 | | 0.4876 | OCT | GloVe | | acuity |  |  |
| 0.75 | 0.5912 | 0.0000 | | 1.0000 | OCT | GloVe | | acuity |  |  |
| 0.9 | 0.5912 | 0.0000 | | 1.0000 | OCT | GloVe | | acuity |  |  |
| 0.1 | 0.7029 | 0.9568 | | 0.5274 | OCT-H | GloVe | | acuity |  |  |
| **Threshold** | **Accuracy** | **Sensitivity** | | **Specificity** | **Method** | **Embedding** | | **Task** |  |  |
| 0.25 | 0.7029 | 0.9568 | | 0.5274 | OCT-H | GloVe | | acuity |  |  |
| 0.75 | 0.7676 | 0.5252 | | 0.9353 | OCT-H | GloVe | | acuity |  |  |
| 0.9 | 0.5912 | 0.0000 | | 1.0000 | OCT-H | GloVe | | acuity |  |  |
| 0.1 | 0.5529 | 1.0000 | | 0.2438 | RNN | GloVe | | acuity |  |  |
| 0.25 | 0.7706 | 0.9712 | | 0.6318 | RNN | GloVe | | acuity |  |  |
| 0.75 | 0.7500 | 0.4029 | | 0.9900 | RNN | GloVe | | acuity |  |  |
| 0.9 | 0.6353 | 0.1079 | | 1.0000 | RNN | GloVe | | acuity |  |  |
|  |  |  |  |  |  |  |  |  |  |  |

CART-Classification and Regression Trees; k-NN-k Nearest Neighbors; OCT-Optimal Classification Trees; OCT-H-Optimal Classification Trees with Hyperplanes; RF-Random Forests; RNN-Recurrent Neural Networks

**eTable 5.** McNemar Test Results for two best performing combinations of featurization technique and binary classification algorithm for all three tasks.

1. **Stroke: GloVe/RNN with BOW/Logistic Regression**

| **Data Split** | $\boldsymbol{\chi}^{\boldsymbol{2}}$ | **p-value** |
| --- | --- | --- |
| 1 | 2.94 | 0.086 |
| 2 | 6.04 | 0.012 |
| 3 | 3.44 | 0.064 |
| 4 | 2.42 | 0.120 |
| 5 | 9.13 | 0.003 |

1. **Location: GloVe/RNN with tf-idf/RF**

| **Data Split** | $\boldsymbol{\chi}^{\boldsymbol{2}}$ | **p-value** |
| --- | --- | --- |
| 1 | 36.13 | 1.85 x 10^-9^ |
| 2 | 20.02 | 7.66 x 10^-6^ |
| 3 | 6.11 | 0.013 |
| 4 | 2.15 | 0.10 |
| 5 | 9.52 | 0.002 |

1. **Acuity: GloVe/RNN with tf-idf/RF**

| **Data Split** | $\boldsymbol{\chi}^{\boldsymbol{2}}$ | **p-value** |
| --- | --- | --- |
| 1 | 50.77 | 1.04 x 10^-12^ |
| 2 | 2.05 | 0.15 |
| 3 | 29.47 | 5.68 x 10^-8^ |
| 4 | 10.02 | 0.0015 |
| 5 | 41.40 | 1.24 x 10^-10^ |

1. **Average results for all tasks**

| **Outcome** | $\boldsymbol{\chi}^{\boldsymbol{2}}$ | **p-value** | **95% CI** |
| --- | --- | --- | --- |
| Stroke Presence | 4.79 | 0.056 | -0.0045:0.12 |
| Location | 14.78 | 0.023 | -0.03:0.08 |
| Acuity | 26.74 | 0.031 | -0.05:0.12 |

**eTable 6.** Performance Comparison of pre-trained Wikipedia GloVe embeddings with the proposed neurology specific embeddings using the RNN classifier across all three predictive tasks.

The values included refer to the average out-of-sample AUC across five splits of the data along with the corresponding 95% confidence intervals. We leveraged the 300-dimensional version, trained on Wikipedia text with 6B tokens and uncased vocabulary of 400K words available here (https://nlp.stanford.edu/projects/glove/). A similar training procedure to the one applied for all other experiments was followed. We replaced out-of-vocabulary words with a randomly initialized UNK token to represent unknown words. This technique was used to represent words, such as encephalomalacia, that were not included in the available Wikipedia-based embeddings.

|  | **Stroke** | **Location** | **Acuity** |
| --- | --- | --- | --- |
| **Wikipedia GloVe** | 0.738 (0.701:0.746) | 0.754 (0.723:0.791) | 0.693 (0.613:0.730) |
| **Neurology GloVe** | 0.961 (0.955:0.967) | 0.976 (0.968:0.983) | 0.925 (0.894:0.955) |

**Supplemental References:**

1. Mikolov T, Sutskever I, Chen K, Corrado GS, Dean J. Distributed representations of words and phrases and their compositionality. *Advances in neural information processing systems*. 2013:3111-3119

2. Pennington J, Socher R, Manning C. Glove: Global vectors for word representation. *Proceedings of the 2014 conference on empirical methods in natural language processing (EMNLP)*. 2014:1532-1543

3. Hastie T, Tibshirani R, Friedman J, Franklin J. The elements of statistical learning: Data mining, inference and prediction. *The Mathematical Intelligencer*. 2005;27:83-85

4. Cover TM, Hart P. Nearest neighbor pattern classification. *IEEE transactions on information theory*. 1967;13:21-27

5. Breiman L. *Classification and regression trees*. Routledge; 2017.

6. Bertsimas D, Dunn J. Optimal classification trees. *Machine Learning*. 2017;106:1039-1082

7. Bertsimas D, Kallus N, Weinstein AM, Zhuo YD. Personalized diabetes management using electronic medical records. *Diabetes Care*. 2017;40:210-217

8. Bertsimas D, Dunn J, Pawlowski C, Silberholz J, Weinstein A, Zhuo YD, et al. Applied informatics decision support tool for mortality predictions in patients with cancer. *JCO clinical cancer informatics*. 2018;2:1-11

9. Bertsimas D, Dunn J, Velmahos GC, Kaafarani HMA. Surgical risk is not linear: Derivation and validation of a novel, user-friendly, and machine-learning-based predictive optimal trees in emergency surgery risk (potter) calculator. *Ann Surg*. 2018;268:574-583

10. Breiman L. Random forests. *Machine learning*. 2001;45:5-32

11. Gurney K. *An introduction to neural networks*. CRC press; 2014.

12. Haykin S. *Neural networks: A comprehensive foundation*. Prentice Hall PTR; 1994.

13. Socher R, Lin CC, Manning C, Ng AY. Parsing natural scenes and natural language with recursive neural networks. *Proceedings of the 28th international conference on machine learning (ICML-11)*. 2011:129-136

14. Hochreiter S, Schmidhuber J. Long short-term memory. *Neural computation*. 1997;9:1735-1780

15. Rao G, Huang W, Feng Z, Cong Q. Lstm with sentence representations for document-level sentiment classification. *Neurocomputing*. 2018;308:49-57
