# Supplementary material for: Machine learning and natural language processing methods to identify ischemic stroke, acuity and location from radiology reports
Source: PLoS One. 2020 Jun 19;15(6):e0234908. doi: 10.1371/journal.pone.0234908 (PMC7304623; doi:10.1371/journal.pone.0234908)
Supplement: Attachment — Submitted filename: Response to Reviewers.docx [file pone.0234908.s003.docx]

**Response Letter to Reviewer Comments on:**

***Machine Learning and Natural Language Processing methods to identify ischemic stroke, acuity, and location from radiology reports.***

We thank the reviewers for their assessment of our work. We have addressed the minor changes suggested in the last revision of our investigation.

In the following, we address the reviewers’ comments point-by-point:

**Reviewer Comments to the Author**

**Reviewer 1**

1. Line 82: “NLP involves” to “ML-based NLP involves”

*We have adjusted the wording accordingly.*

2. Line 83: remove “using specifically tailored machine learning methods”. Many featurization methods are not ML-based. For example, bag-of-words, TFIDF, etc.

*We have removed the highlighted expression.*

3. Line 85: “tf-idf” to “TF-IDF”

*The abbreviation has been adjusted.*

4. Line 86-87: “learn vector representations of word relationships” to “learn a distributed representation for words”

*We have adjusted the expression based on the reviewer’s recommendation.*
